# Leisure time physical activity in Estonian population: adherence to physical activity recommendations and relationships with overweight

**DOI:** 10.1186/s13690-016-0148-6

**Published:** 2016-08-29

**Authors:** Maie Tali, Peeter Lusmägi, Eve Unt

**Affiliations:** 1 Department of Sports Medicine and Rehabilitation, University of Tartu, 1a Puusepa St, 50406 Tartu, Estonia; 2 Sports Medicine and Rehabilitation Clinic, Tartu University Hospital, 1a Puusepa St, 50406 Tartu, Estonia; 3 Estonian Olympic Committee, 102c Pärnu mnt St, 11312 Tallinn, Estonia; 4 Department of Cardiology, University of Tartu, 18 Ülikooli St, Tartu, Estonia

**Keywords:** Physical activity, Leisure time, Estonian population, Adherence to physical activity recommendations, Overweight

## Abstract

**Background:**

Sufficient physical activity (PA) is a key element for the prevention of non-communicable diseases. Considering leisure time physical activity (LTPA), the purpose of the survey was to provide descriptive data for LTPA, find the proportion of the study population meeting the recommended WHO PA criteria, and to detect the possible relationship between LTPA and overweight.

**Methods:**

The National Physical Activity Survey was carried out in autumn 2015 in the Estonian population (*n* = 914) aged 15–69 years. For LTPA assessment, the LTPA domain of IPAQ-L interview version was used. LTPA was analysed in regard to fulfilment of the WHO PA recommendations and in association with BMI.

**Results:**

Seventy-three percent of study participants reported any LTPA in the preceding 7 days. 22 % (26 % of men, 20 % of women) met WHO PA recommendations. 50 % of the study participants were considered overweight (48 % of men, 51 % of women) with BMI > 25.0 kg/m^2^, whereas 20 % of the total study population was obese (BMI ≥ 30.0 kg/m^2^). Lower adherence to WHO PA recommendations was associated with older age in men, and obesity in both men and women.

**Conclusions:**

A strikingly low proportion of people met WHO PA recommendations and a relatively high proportion of overweight people were detected in the study group. Obesity had significant inverse associations with LTPA.

## Background

According to WHO Global Health Risks report, high blood pressure, tobacco use, high blood glucose, sedentary lifestyle, and overweight are the five leading mortality risk factors in the world [1]. These risks are predisposing factors for the development of chronic non-communicable diseases, such as cardiovascular diseases (CVD), diabetes, and cancers [2]. On the other hand, there is augmentative evidence that a healthy lifestyle, including sufficient physical activity (PA), is a key element for the prevention of non-communicable diseases [3–5]. As in other countries in the world, CVD have been the most prevalent cause of early (before 65 years of age) disability and death in Estonia. In 2006, national guidelines for the prevention of CVD were renewed [6] and during the last decade the gradual decline in cardiovascular mortality was achieved [7]. Compared to Estonia’s closest neighbours, Latvia and Lithuania, and other former Soviet Union countries, lower CVD mortality rates have been attained [7]. Still, compared to other European countries, the CVD mortality rates are nearly twice as high or higher [7]. Hence, the modification of CVD risk factors, including lifestyle modification strategies is of utmost importance in achieving further improvements.

The WHO global recommendations on PA for health suggest regular PA [3]. Unfortunately, the PA trends worldwide are inauspicious causing the term “pandemic of physical inactivity” to be introduced [8]. This declaration is in accordance with the latest special Eurobarometer survey on sports and PA, where 59 % of European citizens revealed never or seldom being engaged in PA or played sports [9]. In 2014, data of a health behaviour study, which is carried out every even year since 1990 among the Estonian adult population, indicated that 35.8 % of men and 37.4 % of women are engaged in leisure time PA (LTPA) (the questionnaire includes only one question regarding LTPA: “are you engaged in LTPA lasting at least 30 min per session, and at least 2–3 times per week”) [10]. According to the same study, 57.9 % of adult male population and 52.0 % of female population were overweight (BMI > 25.0 kg/m^2^) [10]. The trends in LTPA have been declining and the proportion of overweight people in the adult population has been increasing over the last decade in the Estonian population [10]. Since the previous data show the low proportions of people engaging in LTPA, there could be a potential reserve for applying interventions for health enhancement. Previous LTPA data of the Estonian population are based on a single question estimate [10], but more specific data are needed for planning interventions.

The purpose of the present survey was to provide descriptive LTPA data and to find the proportion of the study population meeting the recommended criteria of WHO for PA considering LTPA. The secondary purpose was to detect the possible relationship between LTPA and overweight.

## Methods

### Study group

The National Physical Activity Survey was ordered by the Estonian Ministry of Culture. The target population of the survey were permanent residents of Estonia aged 15 to 69 years. According to data of Statistics Estonia, 926,798 people of the required age group lived in Estonia on January 1, 2015 [11]. In the selection of the study group, the proportional model of the population was used, in which all have an equal opportunity of becoming a respondent. Afterwards, the socio-demographic structure of the sample in the breakdown of gender, age, ethnicity and place of residence with the corresponding statistical characteristics of the Estonian population was compared and adjusted if necessary. The initial sample consisted of 1768 respondents, who were visited in their homes by TNS Emor interviewers during September 14 and October 4, 2015. In total, 1004 subjects agreed to participate in the survey. The interviewers were educated to use a tablet, which was pre-programmed with the interview questionnaire. The face-to-face *Tablet Assisted Personal Interviewing* (TAPI) was conducted for data collection. In TAPI, the interview questions appeared on the tablet screen and the answers of a study respondent were inserted in the computer program by an interviewer immediately during the interview, which permits immediate surveillance of the interviewing process. Interviewers asked no guiding questions during the interview.

### Measures

The study participants self-reported their age, body height and weight (for calculation of body mass index – BMI kg/m^2^); educational level (basic or primary, general secondary or vocational, higher); occupation (top manager/manager, professional/specialist/clerk, skilled worker/machine operator/driver; pupil/student; retired; other); monthly income per family member (low – below 300€, average – 301€–550€, over average – over 551€, no income/refused to answer), and place of residence (capital city, town > 35,000 inhabitants, town 3000-35,000 inhabitants, village/rural area).

For the assessment of self-reported LTPA, the LTPA domain of the last seven days interview version of The International Physical Activity Questionnaire (IPAQ-L) was applied [12]. Since the IPAQ-L has not been used in Estonia before the present study, the English version of the questionnaire was translated into Estonian. In the translation process, relevant guidelines and recommendations were taken into account [12]. IPAQ-L was developed and validated at the beginning of the 2000s [13, 14] and is considered a national and international standard for PA surveillance in several countries. The IPAQ-L version provides information about the time spent in and intensity of PA for different domains (occupation, transportation, home, leisure time). Compared to other domains, IPAQ studies have shown the highest validity for LTPA [15–17]. In the present study, the LTPA domain (6 items) of IPAQ-L was used. Respondents were asked to report the frequency and duration of walking, moderate and vigorous intensity LTPA performed for at least 10 min per LTPA session during the last 7 days. Moderate activities were defined as activities with a temperate increase in respiration and heart rate (for example: brisk walking, Nordic walking, bicycling at a regular pace, swimming at a regular pace, doubles tennis). Vigorous activities were defined as activities with a significant increase in respiration, heart rate, and sweating (aerobics, running, fast bicycling, or fast swimming). Energy expenditure was expressed as metabolic equivalents of task (MET) multiplied by the time in minutes per week for walking, moderate and vigorous activity, and for the total LTPA (MET minutes per week). One MET corresponds to 3.5 ml O_2_ × kg^−1^ × min^−1^.

### Data processing

For LTPA items (walking, moderate and vigorous), weekly and average time per day was calculated. 90 subjects were excluded due to confounding or missing data (reporting LTPA in more than 7 days a week, or refused). The final sample for LTPA prevalence analysis was 914 (373 men).

Age was categorized into five groups (15–24 years, 25–34 years, 35–49 years, 50–64 years, and 65–69 years) for both genders.

According to BMI, the study subjects were categorized as ‘underweight’– BMI < 18.5, ‘normal weight’ – BMI 18.5–24.9, ‘overweight’ – BMI 25.0–29.9, and ‘obese’ – BMI >30.0 [4]. Due to the unbalanced men/women ratio 1:17 in ‘underweight’, this group is not presented in the figures. For subjects aged 15–17 years, population-based age-adjusted criteria for BMI categorization were applied [18].

According to WHO recommendations, at least 150 min of moderate-intensity aerobic PA or 75 min of vigorous-intensity PA throughout the week is recommended for adults aged 18–64 years [3]. For adolescents, the WHO PA recommendation encompasses accumulation of at least 60 min of moderate-vigorous PA in every day of the week [3]. As there are no separate recommendations for LTPA and former research has shown that European adults are mostly physically active during leisure time [9], the WHO PA recommendations were used as a criterion for achieving a sufficient LTPA level. The latter approach has been implemented in earlier studies concerning LTPA [16, 19]. Regarding achievement of WHO PA recommendations, a dichotomized variable (meeting/not meeting) was created. To meet regularity and accumulation of moderate to vigorous LTPA in adults, the variable had to meet the following criteria: 1) 30 min of moderate LTPA per day on at least 5 days of the week; 2) accumulating 150 min of moderate LTPA per week with LTPA reported on at least 3 days of the week; 3) accumulating 25 min of vigorous LTPA on at least 3 days of the week; 4) a combination of moderate and vigorous LTPA on at least 3 days of the week with a total duration 150 min/week. In adolescents, accumulation of at least 60 min of moderate-vigorous LTPA in every day of the week was regarded as adherence to PA recommendations [3].

### Statistical analysis

Statistical analyses were carried out with the Statistical Package for the Social Sciences – SPSS, 22 (IBM, Armonk, NY, USA). To characterize the study sample, descriptive data – n, percentage (%), mean, SD, median, 25^th^ and 75^th^ percentile – were calculated. All LTPA distributions were skewed to the right. The unpaired *t*-test for parametric data and the Mann–Whitney *U*-test for nonparametric data were used to determine the differences between the groups. The chi-square (*χ*2-test) test was used to determine significant differences in proportions between the groups. Multinomial logistic regression analysis was conducted to reveal associations between achievement of PA recommendations and age groups, and BMI categories. In age groups the youngest group and in BMI categories the normal BMI category were set as reference groups. For all statistical analyses, the 0.05 level of significance was applied.

## Results

The subjects’ mean age was 39.7 ± 15.3 years and 44.0 ± 15.9 years for men and women, respectively. In the final study group, there were more women than men and women were slightly older than men.

Seventy-three percent of the study participants reported LTPA in the preceding 7 days (Table 1). Walking was the most frequently reported LTPA, while moderate-vigorous LTPA was reported at a considerably lower rate. Men reported significantly more vigorous LTPA than women (Table 1).

**Table 1:** Leisure time physical activity duration and energy expenditure and the prevalence of people reporting respective leisure time physical activity according to gender. Leisure time physical activity survey of Estonian population in 2015

	Men **(** *n* = 373)		Women (*n* = 541)		Total study group (*n* = 914)	
	Median (P 25^th^, P 75^th^)	%	Median (P 25^th^, P 75^th^)	%	Median (P 25^th^, P 75^th^)	%
LT Walking (min/day)	8.7 (0; 34.6)	58.2	17.1 (0; 60.0)	61.6	11.4 (0; 42.9)	61.1
Moderate LTPA (min/day)	0 (0; 15.0)	33.2	0 (0; 9.3)	33.3	0 (0: 12.8)	33.9
Vigorous LTPA (min/day)	0 (0; 17.1)	35.1	0 (0; 0) ***	23.3	0 (0; 5.9)	27.4
LT Walking (MET min/week)	198.0 (0; 800.3)		396.0 (0; 1097.2)		264.1 (0; 990.0)	
Moderate LTPA (MET min/week)	0 (0; 420.0)		0 (0; 260.8)		0 (0; 360.0)	
Vigorous LTPA (MET min/week)	0 (0; 960.0)		0 (0; 0) ***		0 (0; 320.0)	
Total LTPA (MET min/week)	891.0 (0; 2415.0)	70.2	760.0 (0; 2079.0)	74.3	792.0 (0; 2310.8)	72.6

Note: ***p < 0.001 women compared to men of the respective group

P 25^th^, P 75^th^ – 25 ^th^ and 75 ^th^ percentile

*LT* leisure time

*LTPA* leisure time physical activity

The characteristics of the study group and the prevalence of people reporting LTPA, as well achievement of WHO PA recommendations in age groups and sociodemographic groups are presented in Table 2. The prevalence of reporting LTPA was higher in younger age groups. People with higher income and higher education reported participation in vigorous LTPA more often than people with low income and basic education. Among small-town male inhabitants, the higher prevalence of walking but lower prevalence of moderate LTPA was observed compared to capital residents; among women, the prevalence of walking was higher in village residents and the prevalence of moderate LTPA was lower in small-town residents. Compared to top managers, the prevalence of any LTPA was considerably lower in male skilled workers, whereas the prevalence of walking in LT was lower among female skilled workers. Considering WHO PA recommendations for weekly LTPA, 22 % of study participants met the criteria in total – 26 % of men and 20 % of women. The highest prevalence of people meeting WHO PA criteria was observed in men aged 15–24 years, whereas in other groups the prevalence was below 30 % with the lowest in men aged 65–69 years. Compared to the youngest age group of men, the WHO PA criteria were met by a considerably lower proportion of men older than 25 years, and by women of the youngest age group (p < 0.01). Among men, a significantly lower proportion of skilled workers and retired men compared to top managers met the WHO PA criteria. In contrast, the proportion of male students meeting the criteria was considerably higher compared to top managers.

**Table 2:** The characteristics of the study group and the prevalence (%) of study group subjects reporting leisure time physical activities, and adherence to WHO physical activity recommendations. Leisure time physical activity survey of the Estonian population in 2015

	Men (*n* = 373)					Women (*n* = 541)				
	%	Walking %	Moderate LTPA %	Vigorous LTPA %	Adherence to WHO PA recommendations %	%	Walking %	Moderate LTPA %	Vigorous LTPA %	Adherence to WHO PA recommendations %
Age groups										
15–24 years	18.8	78.6	50.0	65.7	45.7	13.9	66.7	38.7	40.0	24.0
25–34 years	22.8	58.8**	37.6	34.1***	27.1*	17.6	68.4	30.5	26.3	17.9
35–39 years	30.6	50.9***	35.1	29.8***	24.6**	27.9	60.3	30.5	23.8*	19.2
50–64 years	21.2	50.6***	25.3**	12.7***	15.2***	29.1	53.2**	34.8	17.7***	20.9
65–69 years	6.6	52.0*	12.0***	20.0***	12.0***	11.5	29.0***	33.9	11.3***	14.5
Education										
Basic/primary^**a**^	11.3	45.5	27.3	12.1	18.2	7.3	27.3	33.7	10.0	13.3
Secondary/vocational^**a**^	64.2	38.3	29.8	28.7**	24.5	58.4	58.9	31.5	17.4	19.5
Higher	24.5	68.1	40.3	38.9**	23.6	34.3	64.8	37.3	28.9*	20.7
Place of residence										
Capital	34.3	28.9	37.5	37.5	31.3	31.1	29.2	35.7	26.2	18.8
Big town, >35,000 inhabitants	15.8	28.9	40.7	40.7	33.3	18.6	37.6	36.6	20.8	27.7
Small town, 3000–30,000 inhabitants	16.9	68.3***	30.2**	28.6	23.9	19.2	34.6	28.8**	24.0	19.2
Village	33.0	42.3	28.2	20.5*	21.1	31.1	50.0***	38.7	21.3	16.7
Income										
Low (<300 €)	14.7	49.1	27.3	16.4	21.8	18.1	44.9	18.6	11.2	18.8
Average (301–550 €)	23.9	49.4	23.6	33.7*	23.6	34.0	35.9	38.0	20.6*	19.7
High (>550 €)	35.4	38.6	45.5	37.9***	30.3	22.4	37.2	31.4	29.8***	16.5
No income/refused	26.0	36.1	35.1	36.1**	25.8	25.5	38.4	31.9	29.7**	23.2
Occupation										
Top manager	10.2	76.3	47.4	42.1	31.6	15.0	63.0	35.8	32.1	21.0
Professional/specialist	18.2	66.2	45.6	42.6	30.9	22.5	68.9	36.0	21.3	18.9
Skilled worker	28.2	48.6***	20.0**	19.0***	15.2***	17.2	47.3*	34.4	19.4	20.4
Pupil/student	12.8	81.3	56.3	64.6*	45.8*	8.5	78.3	45.7*	54.3*	28.3
Retired	12.6	46.8	51.1	17.0*	21.3***	18.1	58.2	18.4*	8.2***	16.3
Other/Missing value	18.0	46.9***	13.4	30.0	4.5***	18.7	59.8	23.7	18.8	15.8

Note: ^**a**^ – pupils and students were excluded due to misclassification of education level

*p < 0.05; **p < 0.01; ***p < 0.001 compared to the reference group (age group 15–24 years, basic/primary education, capital, low income, top manager) of the respective gender

*PA* physical activity

*LTPA* leisure time physical activity

**Fig. 1:**
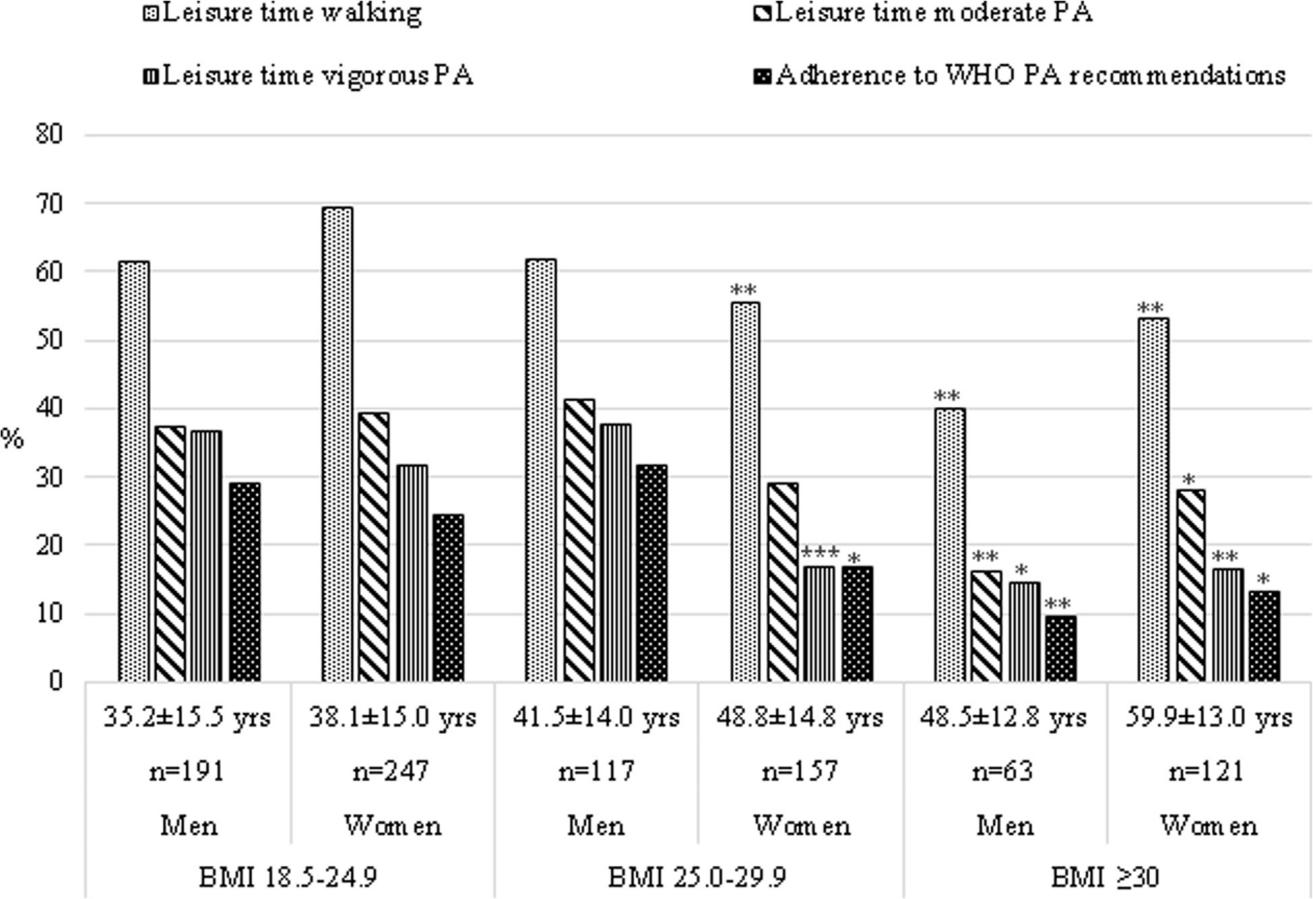
Proportion (%) of study group subjects participating in leisure-time physical activities and adherence to the WHO physical activity criteria, according to BMI groups. Note: *p < 0.05; **p < 0.01; ***p < 0.001 compared to the respondents in the normal body weight group (BMI 18.5–24.9 kg/m^2^ in adults and BMI in normal age adjusted range in adolescents) of respective gender. PA – physical activity

The mean BMI was 25.7 ± 4.7 kg/m^2^ and 26.2 ± 5.7 kg/m^2^ for men and women, respectively. 50 % of the study participants were considered overweight (48 % of men and 51 % of women), whereby 20 % of the total study population was obese (17 % of men and 22 % of women). The prevalence data of LTPA and adherence to WHO PA recommendations according to BMI categories are presented in Fig. 1. Compared to people with BMI in the normal range, a significantly lower proportion of overweight women as well obese men and women reported LT walking or vigorous LTPA and hence, there was a considerably lower prevalence of people in those groups who met the recommended WHO PA criteria. As an exception, in men with BMI in range 25.0–29.9 kg/m^2^, the proportion meeting WHO PA recommendations was the highest (32 %) compared to other BMI groups. In ‘underweight’ women (not presented in Fig. 1), adherence to PA recommendations did not significantly differ from the ‘normal weight’ group of the respective gender (28 % *vs* 24 %, respectively).

Lower adherence to WHO PA recommendations was associated with older age in the total study group, which was mainly attributed to men, and obesity in both men and women (Table 3).

**Table 3:** Associations between achievement of WHO physical activity recommendations and age, and body mass index [adjusted OR (95 % CI)]. Leisure time physical activity survey of the Estonian population in 2015

		Achievement of WHO physical activity recommendations		
		Men (*n* = 373)	Women (*n* = 541)	Total (*n* = 914)
Age groups (OR adjusted for BMI, education level, occupation and place of residence)				
	15–24 years (reference group)	1.0	1.0	1.0
	25–34 years	2.37 (1.05–5.36)*	1.59 (0.69–3.66)	2.01(1.14–3.53)*
	35–49 years	2.49 (1.13–5.50)*	1.29 (0.57–2.91)	1.88 (1.08–3.27)*
	50–64 years	4.30 (1.12–5.50)*	1.02 (0.45–2.29)	1.92 (1.08–3.43)*
	65–69 years	5.23 (1.30–21.2)*	1.53 (0.56–4.19)	2.64 (1.21–5.73)*
BMI categories (ORs are adjusted for age, education level, occupation and place of residence)				
	18.5–24.9 (reference group)	1.0	1.0	1.0
	25.0–29.9	0.71 (0.41–1.20)	1.67 (0.97–2.86)	1.07 (0.74–1.54)
	>30.0	2.55(1.01–6.46)*	2.20 (1.15–4.21)*	2.14 (1.27–3.61)**
	<18.5	-	0.91 (0.28–3.00)	-

Note. *p < 0.05, **p < 0.01 compared to the reference group

Data for underweight men’s group are not presented (includes one subject)

## Discussion

In the present study, we focused on the description of LTPA among the Estonian population and adherence to WHO PA recommendations considering LTPA as well as on possible associations between LTPA and overweight. The study results revealed that 73 % of the study participants reported LTPA in the preceding 7 days, whereas 22 % (26 and 20 % of men and women, respectively) met WHO PA recommendations. Half of the study group participants had BMI > 25.0 kg/m^2^ and were thus considered overweight, and 20 % were obese. In the obesity group, considerably lower proportion of people reported LTPA and hence, the adherence to WHO PA recommendations was lower (in men as well as women) compared to the normal body weight group.

The prevalence of study respondents reporting any LTPA in Estonia (73 %) is roughly similar with other European Union countries (73.1 %) [20] and the US (70.8 %) [21]. Although nearly three-fourths of the study participants’ reported LTPA, the amount of LTPA was rather modest, with median time per day for walking 11 min and moderate-vigorous LTPA 0 min. The latter means that at least half of subjects did not report any moderate-vigorous LTPA. Previous studies have demonstrated a wide variety in the LTPA amounts across countries. Calculations of energy expenditure for LTPA activities allow the comparisons between the countries. We acknowledge that the comparisons may be imprecise due to different methods used for LTPA assessment. Regardless, Estonians’ LTPA level (median 13.2 MET-hours/week) was, according to previously reported data, slightly higher than in southern European countries (<10 MET-h/week in Greece, Italy, Spain, and Portugal), in comparable range with the population of Germany (12.7 MET-h/week) and the United Kingdom (16.0 MET-h/week), but falling greatly behind the Netherlands (21.0 MET-h/week), Finland (21.5 MET-h/week), Austria (23.0 MET-h/week), and Sweden (24.0 MET-h/week) [20]. In previous studies, the north to south decline in LTPA has been described [20, 22], but despite the Nordic geographic location of Estonia, the LTPA is rather comparable to southern countries, implying that Estonian residents are physically more inactive than residents in neighbouring countries.

The majority of the present study population reported LTPA. However, the amount, intensity and regularity sufficient to meet the PA recommendations for weekly PA was achieved only in a fifth of them, whereas compliance was somewhat higher in men compared to women. The finding in regard to the gender difference, of men being more active than women is in accordance with previous studies [16, 19, 20, 23]. The finding that higher proportions of those engaged in vigorous LTPA are mostly men and rather people in younger age groups is also compatible with previous results [20, 24]. Compared to Estonian data from 2014, whereby 35.8 % of men and 37.4 % of women reported participation in LTPA [10], the LTPA prevalence is lower in the current survey and more men than women were sufficiently active in LT. Suspected reasons of this discrepancy could be the difference in LTPA data collection on the one hand, and stricter criteria applied for PA recommendation adherence in the latter survey on the other hand.

Many studies have reported the age-related decline in physical activity [19–21]. Among the present study population, the group of the youngest men differed distinctly with the highest prevalence of those meeting WHO PA recommendations, and among men, the gradual ageing-dependent decline in the prevalence of sufficiently active persons was demonstrated. In women, the same tendency was observed but since the prevalence of those meeting PA recommendations was low in the youngest age group, the decline did not seem as drastic as in men.

In addition, considering the socio-demographic indices of the study population the vigorous LTPA was more often reported by people with higher education and higher income, whereas walking was more often reported by residents of smaller towns and villages. Regardless, these differences in LTPA patterns did not significantly affect the adherence to WHO PA recommendations in people with different levels of education and income or place of residence. However, in male skilled workers the prevalence of any LTPA and hence adherence to PA recommendations was considerably lower compared to the reference group of top managers. These findings are in concordance with previous studies [20, 23]. The probable reason for lower LTPA in men with a blue-collar occupation is the high level of work-related PA, which precludes the participation in LTPA. Hence, in subgroup of people with a blue-collar occupation the separately analysed LTPA may significantly underestimate the PA level and the assessment of work-related PA is essential.

Concerning body weight, the inverse relation between LTPA and overweight is well documented [20, 23, 25, 26]. As an exception in the present study, men with BMI in the overweight range were equally active in LT compared to men with normal body weight. It could probably be explained by physically active men in this group having higher BMI due to larger muscle mass rather than a higher proportion of adipose tissue. According to our data, obesity was a factor that is associated with lower adherence to WHO PA recommendations. The current PA recommendations include only engagement in moderate and vigorous PA, but this could be hard to accomplish by obese and/or older people. Considering the LTPA pattern, walking was the most prevalent activity in the present study population, whereas moderate-vigorous LTPA was reported by a considerably lower proportion of respondents, particularly in overweight women, and obese men and women. The inverse correlation between BMI and vigorous PA (including LTPA) has also been shown in a Norwegian study [15]. According to Loprinzi et al. [27], there is growing evidence that light-intensity PA encompasses similar health enhancing effects as moderate-vigorous PA. Albeit the amount of time needed to achieve those effects is at least twice as much (≥300 min/week) [27] as the current PA recommendation, this could be more suitable for obese and older people.

The strength of the present study is the stratified and randomly selected study population. In the final study group, there were more women than men and women were slightly older than men, but the study sample was representative of the Estonian population structure. Nevertheless, we acknowledge that the present study has several limitations. The BMI was calculated based on height and weight stated by the study respondents. The BMI calculated in this way may be overestimated although this effect is considered rather modest [28]. Typically, participants overestimate height and underestimate weight resulting in a BMI that is, on average, 1 kg/m^2^ lower than when height and weight are actually measured [29]. Thus, the proportion of overweight people could be even higher in the present study. Another limitation is the questionnaire-based evaluation of LTPA. Although the interview-based IPAQ-L version questionnaire has shown better validity than self-reported questionnaires [30], there is critical evidence in the literature about the overestimation of PA, especially in PA related to work and household domains [16, 17], which we also encountered in our data. Compared to other IPAQ-L domains, LTPA is considered the most valid measure in categorizing the population as physically active or sedentary [15, 17]. Recently published data in adults demonstrated a distinct discrepancy in PA level outcomes when different methods were used in PA evaluation, whereas self-reported PA showed systematic over-reporting of moderate-vigorous PA up to 10 times compared to accelerometer-based studies [31]. Hence, the over-reporting issue of self-reported PA with IPAQ-L cannot be overlooked. To overcome the over-report issues, stricter criteria for the calculation of the achievement of PA recommendations were applied in the present survey, where the accumulated time amount, intensity, as well the regularity of PA were taken into account. We acknowledge that the objectively measured PA using accelerometers would have given a more precise estimation of the PA level, but the main concerns in carrying out the present study were the cost and feasibility, and the objective PA measurement was not considered. Hence, before planning interventions for certain groups at the risk of physical inactivity, more precise PA monitoring than self-report should be taken into consideration.

## Conclusion

In summary, the majority of the present study population reported LTPA. However, only fifth of them were sufficiently physically active to meet the WHO recommendations for weekly PA. Lower adherence to WHO PA recommendations was associated with older age in men, and obesity in both men and women. Although the purpose of the present descriptive study was not to explore the whole spectre of reasons influencing LTPA, the results of the present survey provide useful baseline data about LTPA and adherence to PA recommendations as well as associations between LTPA and overweight in the Estonian population. The strikingly low proportion of people in relation to sufficient LTPA demands future research in this area to promote increasing PA.

## Abbreviations

BMI, body mass index; CVD, cardiovascular diseases; IPAQ-L, The International Physical Activity Questionnaire long form; LTPA, leisure time physical activity; MET, metabolic equivalent of task; PA, physical activity; TAPI, Tablet Assisted Personal Interviewing
